# Psychological Distress Among Asian Indians and Non-Hispanic Whites in the United States

**DOI:** 10.1089/heq.2021.0159

**Published:** 2022-07-05

**Authors:** Zasim Azhar Siddiqui, Usha Sambamoorthi

**Affiliations:** ^1^ Department of Pharmaceutical Systems and Policy, West Virginia University School of Pharmacy, Robert C. Byrd Health Sciences Center, Morgantown, West Virginia, USA.; ^2^ Department of Pharmacotherapy, College of Pharmacy, Texas Center for Health Disparities, University of North Texas Health Science Center, Fort Worth, Texas, USA.

**Keywords:** Asian Indian, Kessler (K6) psychological distress scale, National Health Interview Survey, psychological distress

## Abstract

**Introduction:**

The prevalence of psychological distress (PD) among Asian Indians is unknown. This study estimated and compared moderate–serious PD in Asian Indians and non-Hispanic Whites (NHWs) in the United States.

**Methods:**

We used a cross-sectional design using the National Health Interview Survey (2012–2017). Adult (age >18 years) NHWs and Asian Indians (*N*=2,218) were included. PD was measured using the six-item Kessler (K6) scale. We used multivariable logistic regression to determine the association of Asian Indian ethnicity with PD.

**Results:**

In the analysis, 19.9% of NHWs and 11.0% of Asian Indians reported moderate–serious PD. Asian Indians were less likely to report PD in both unadjusted (unadjusted odds ratio=0.50; 95% confidence interval [CI]=0.42–0.58) and fully adjusted (adjusted odds ratio=0.7; 95% CI 0.59–0.82) models.

**Conclusions:**

Asian Indians had a lower prevalence of PD than NHWs, likely due to multiple protective factors such as high socioeconomic status and lower multimorbidity.

## Introduction

Psychological distress (PD) is a risk indicator for common mental health disorders in a community, and it is widely used in population health and epidemiological studies.^1–3^ It is defined as a set of painful mental and physical symptoms that are associated with normal fluctuations of mood in most people. In some cases, however, psychological distress may indicate the beginning of major depressive disorder, anxiety disorder, schizophrenia, somatization disorder, or a variety of other clinical conditions. It is assessed by many putative self-report measures of depression and anxiety.^4^

The prevalence of PD has remained stable in the United States for the last two decades.^5–7^ Recent studies reported a mean prevalence of serious PD in the range of 2.6% to 3.6%,^5–7^ whereas the prevalence of moderate PD was reported as 15.1%.^6^

PD is influenced by a multitude of factors that could act as risk factors for PD^8–15^ or protective against PD.^16–24^ The risk factors for PD include chronic health conditions, physical functional impairment, discrimination, and high work- and education-related stress.^8–15,25^ The protective factors include high education, high income, employment status, high social support, health insurance, and physical activities that are associated with lower PD.^16–24,26–29^ Immigration status may also act as a protective factor (healthy immigrant effect), suggesting that first-generation immigrants usually have better physical and mental health than the natives of host countries.^30–32^

Variations in PD across different racial and ethnic groups are of particular interest because of systematic differences in factors that may protect against PD or increase the risk of PD. Racial minorities experience varying levels of stress exposure, but have abilities and resources to cope with them.^24^ For example, racial/ethnic minorities can face additional stressors such as perceived racism, stigmatization, and discrimination that can increase the risk of PD.^33^ On the other hand, one's racial/ethnic identity itself may be used as a coping factor, which in turn may become a protective factor against PD.^34^

While education, income, employment, old age, male sex, and social support are well-documented protective factors across all racial/ethnic groups, chronic diseases and disability explain the differences in the prevalence of PD among racial/ethnic groups.^17,18,21,24,35,36^ However, the degree of protection and magnitude of risk may vary across racial/ethnic groups. For example, the effect of chronic diseases in developing PD is highest among Native Americans, Blacks, and Hispanics.

In comparison, the least impact of chronic diseases is seen among non-Hispanic Whites (NHWs) and Asian Americans.^21,24^ Moreover, studies show that differences in PD persist even after controlling for the risk and protective factors for racial/ethnic groups.^17,24^

Most studies on PD, by race/ethnicity, focus on NHWs, African Americans, or Hispanics.^37–43^ Even when other racial/ethnic minorities are examined, the studies combine the racial groups. Researchers generally combined all the Asian American ethnicities into one group (e.g., Asian Indian, Chinese, Filipino, Japanese, Korean, Vietnamese), whereas research shows challenges in grouping all the Asian American ethnicities together due to their disparate socioeconomic status.^32,44^

Existing studies have generalized the cultural background of Asian Americans and treated them as one racial group.^21,45^ One recent study examined PD among the racial subgroups of Asian Americans.^20^ This study observed that despite some shared cultures, the Asian races are culturally diverse, and PD among these subgroups can be significantly different.^20^ However, it only included Chinese, Filipino, Vietnamese, Korean, and Japanese Asian American groups and did not include Asian Indians. Besides, this study included data from only one U.S. state.

Asian Indians compose 19% of the Asian American population in the United States.^46,47^ Asian Indians have characteristics that serve as protective factors against PD. For instance, Asian Indians in the United States have high educational attainment, lower unemployment, lower poverty rate, and higher social support through marriage than the general population.^48^ Studies have also shown that Asian Indians retain a strong culture, ethnic identity, and traditional family structure at home while adapting to the U.S. culture and propriety outside the home.^49,50^

Besides the protective factor, Asian Indians are exposed to multiple risks that could lead to PD. For instance, the Asian Indian population in the United States is younger,^48^ reports a high incidence of discrimination,^51^ and has a high prevalence of chronic diseases, such as diabetes and coronary heart disease, and perceived discrimination for accessing health care services.^52,53^

Existing literature shows that Asian Indians have several protective and risk factors that could help them cope or develop/exaggerate PD. No study has evaluated PD in Asian Indians in the United States. Thus, the objective of this study is to assess PD in the Asian Indian population and compare it with the NHW population in the United States using nationally representative data.

## Methods

### Study design

This study used a cross-sectional design using National Health Interview Survey (NHIS) data from 2012 to 2017. The study was performed using NHIS public-use files consisting of deidentified data, hence it does not require ethics committee approval.

### Data source

NHIS is an annual cross-sectional survey designed to monitor the health of the civilian noninstitutionalized population of the United States.^54,55^ It is conducted by the National Center for Health Statistics and was initiated in 1957. The NHIS collects data on topics related to demographics, health insurance, health care access, health care utilization, health conditions, and behavioral risk factors.

In this study, we used the core survey—household, family, and sample adult components. The sample design involves multistage clustering, stratification, oversampling of specific groups, and use of survey weights to adjust for nonrespondents.

### Study sample

Our study sample consisted of all NHWs and Asian Indian adults (age ≥18 years) who responded to the sample adult survey and did not have any missing value on the PD measure, as defined by the six-item Kessler (K6) scale. We pooled NHIS data from 2012 to 2017 to ensure an adequate sample size for the Asian Indian subgroup.

**Appendix Figure A1.**
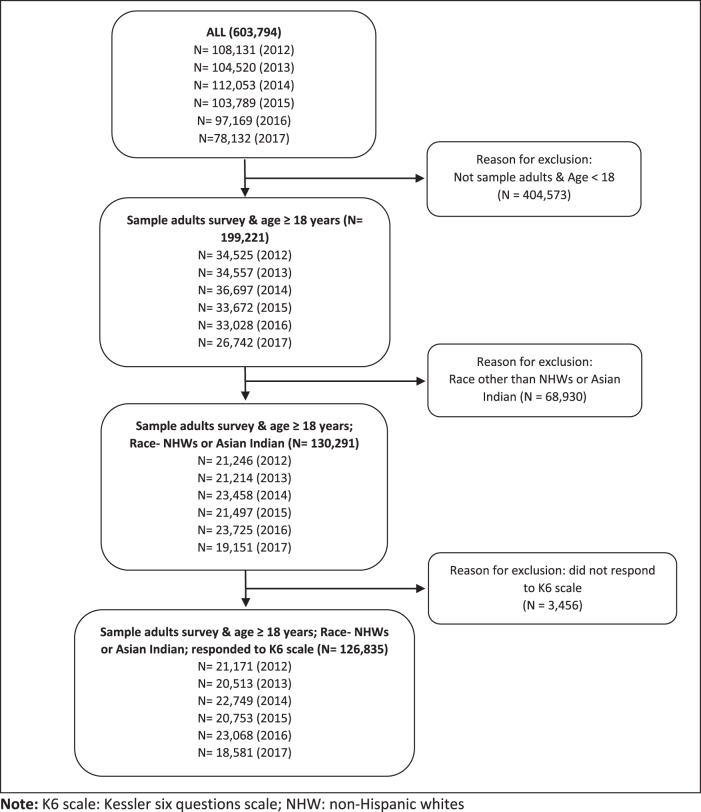
Study sample selection: National Health Interview Survey, 2012–2017. K6, six-item Kessler; NHWs, non-Hispanic Whites.

The steps for the study sample selection are described in Appendix Figure A1. The final sample consisted of 126,835 participants (2,218 Asian Indians and 124,617 NHWs).

### Measures

#### Dependent variable: moderate–serious nonspecific PD

The topic of PD was introduced into the survey in 1997.^56^ NHIS uses the K6 questions, commonly known as the K6 scale, to identify PD. This scale was developed by Kessler et al. for use in the core survey of the NHIS.^56^ The scale measures nonspecific PD rather than disorder-specific distress. The K6 scale in the NHIS contains six questions about the participant's mental state in the last 30 days. These questions asked subjects how often (in the last 30 days) they felt sad, nervous, restless/fidgety, hopeless, everything was an effort, and worthless. These items are rated on a five-point Likert scale from “none of the time” (response=0) to “all of the time” (response=4), with the summary score ranging from 0 to 24.

Conventionally, the K6 scale uses the cutpoint score of K6≥13 to identify serious PD. Prochaska et al. determined and validated the subthreshold cutpoint to distinguish between no or low distress (K6<5), moderate distress (5 ≥ K6<13), and serious distress (K6≥13).^57^ Due to the low prevalence of serious PD, the added moderate threshold in the K6 scale helps to identify participants with significant, but not serious, PD.

In this study, we used the K6 scale score of ≥5 as a dependent variable to identify the sampling population with moderate–serious PD. We combined the two cutpoints due to the low prevalence of serious PD, especially considering the smaller sample size of the Asian Indian population in the United States.

#### Key independent variable: Asian Indians versus NHWs

Race/ethnicity was used as a key independent variable and classified as NHWs and Asian Indians to assess moderate–serious PD between the two groups. Participants were categorized as NHWs and Asian Indians based on their responses to the NHIS questions on (1) origin (Hispanic, Latino, or Spanish origin) and (2) race.

Individuals who responded no to the first question and selected White for race were categorized as NHWs. Individuals who responded no to the first question and selected Asian American and the subcategory Asian Indian for the second question were categorized as Asian Indians. In this study, we used only the Asian Indian race as a key independent variable as PD in people of other races and ethnicities, including Asian Americans and their subgroups, has been studied.^17,20,21,24,58^

#### Other independent variables

For the other independent variables, we used the individual characteristics that are known to be associated with PD based on published literature.^17,59–66^ We used biological factors such as age (18–39, 40–49, 50–64, or ≥65 years) and sex (male and female). Marital status was used to determine the respondent's social support. Socioeconomic status was determined using education level, employment status, and income level. For determining access to health care, we used insurance status (insured and not insured).

We also included the number of chronic diseases and conditions as no diseases, one disease, and two or more diseases. We used the race-adjusted body–mass index (BMI) to account for differences in the classification of overweight and obesity in Asian Indians and NHWs, as recommended by the World Health Organization guidelines.^67^ Physical exercise and activity were recorded as daily, weekly, monthly, or never.

The existing literature shows a bidirectional relationship of PD and behavioral characteristics, such as between smoking and PD^62–64^ as well as between alcohol use and PD.^65,66^ We included participants' smoking status (never, past, or current smoker) and their alcohol use status (never, past, or current alcohol user) to observe the effects of these behaviors on PD. The geographical region (Northeast, Midwest, South, and West) and the NHIS (2012–2017) were used as external factors.

### Statistical analyses

Unadjusted differences in moderate–serious PD between NHWs and Asian Indians were examined using the Rao–Scott chi-square test. Multivariable logistic regression was used to examine the association between race/ethnicity and moderate–serious PD. In the regression model, independent variables were added in sequential blocks to observe their effect on the dependent variable.

The first model was the unadjusted model with only race/ethnicity as an independent variable. In model 2, we added biological factors, age and sex, to the unadjusted model. In model 3, we added education as it is highly protective against PD. In model 4, we added the rest of the protective factors observed in the literature, which include marital status, socioeconomic status, health insurance, and physical activity. In model 5, we included the risk factors for PD, which included race-adjusted BMI, number of chronic diseases, and participants' smoking and alcohol use status. In model 6, we added geographical regions and NHIS years.

Parameter estimates from regression were transformed to odds ratios (ORs) and their confidence intervals were determined at 95%. The statistically significant level was set at *p*≤0.05. All analyses incorporated the strata and weights provided by the NHIS to account for the complex survey design. All analyses were performed using SAS 9.4 (SAS Institute, Inc.).

## Results

### Sample characteristics

Based on the study criteria, data on 2,218 Asian Indians and 124,617 NHWs were analyzed. About half were women (51.4%) and younger than 50 years (50.1%). The majority were married (63.8%), with more than high school education (66.9%), were employed (60.1%), and had health insurance (91.2%). Nearly one in three (62.5%) reported at least one chronic condition.

Appendix Table A1 describes the characteristics of the sample in detail.

### Description of characteristics of Asian Indians and NHWs

We found that a high percentage of the Asian Indian population was younger (74.2% participants were <50 years old) compared with NHWs (49.6% participants were <50 years old). In comparison with NHWs, Asian Indians reported a higher percentage of marriage (77.1% vs. 63.6%), college education (73.20% vs. 34.5%), employment (68.50% vs. 60%), and income above 400% Federal Poverty Level (FPL) (54.40% vs. 42.9%).

The prevalence of chronic diseases was significantly higher in NHWs compared with Asian Indians as 63.1% NHWs reported one or more chronic diseases, whereas only 38.5% of Asian Indians reported one or more chronic diseases. Similarly, NHWs showed a higher prevalence of current smoking status (17.7% vs. 4.8%) and alcohol use (69.9% vs. 44.1%). A higher percentage of Asian Indians were obese compared with NHWs (48% vs. 28%).

Table 1 describes differences in demographics, lifestyle, socioeconomic status, behavioral characteristics, and health status by race/ethnicity.

**Table 1.** Description of Sample by Racial/Ethnic Characteristics of Adults (≥18 Years) Using the National Health Interview Survey, 2012–2017

Sample characteristics	NHWs		Asian Indians		*p*
	*N* (124,617)	Wt. %	*N* (2,218)	Wt. %	
Moderate–serious PD (K6≥5)					<0.001
Moderate–serious PD	25,827	19.9	267	11.0	
No PD	98,790	80.1	1,951	89.0	
Serious PD (K6≥13)					<0.001
Serious PD	4,553	3.4	31	1.00	
No PD	120,064	96.6	2,187	99.0	
Sex					0.015
Women	67,514	51.5	1,000	48.0	
Men	57,103	48.5	1,218	52.0	
Age in years					<0.001
18–39	37,116	33.6	1,332	53.5	
40–49	17,956	16.0	402	20.7	
50–64	34,458	27.8	308	17.8	
≥65	35,087	22.5	176	8.0	
Marital status					<0.001
Married	65,643	63.6	1,509	77.1	
Widow, separated, or divorced	34,578	17.8	160	5.5	
Never married	24,165	18.5	546	17.3	
Education					<0.001
Less than high school	10,812	8.3	93	5.3	
High school	31,133	24.9	189	10.0	
Some college	40,421	32.0	237	11.3	
College	41,949	34.5	1,693	73.2	
Poverty status					<0.001
<100% Federal Poverty Level (FPL)	13,384	8.3	247	8.4	
100 to <200%	19,619	13.7	235	9.9	
200 to <400%	34,177	27.0	404	19.7	
≥400%	47,614	42.9	1,159	54.4	
Employment					<0.001
Employed	70,578	60.0	1,544	68.5	
Unemployed	53,989	40.0	673	31.5	
Health insurance					0.661
Insured	113,558	91.2	2,029	91.5	
Uninsured	10,722	8.5	182	8.2	
Physical activity/exercise					<0.001
Daily exercise	8,572	7.0	159	7.2	
Weekly	44,802	37.8	950	41.4	
Monthly, yearly, or never	67,424	52.5	1,089	50.3	
Unable to exercise	2,862	1.8	5	0.3	
Race-adjusted BMI					<0.001
Underweight and normal	44,386	35.7	707	29.7	
Overweight	41,677	33.4	466	21.2	
Obese	34,932	28.0	1,020	48.0	
No. of chronic diseases					<0.001
No	42,419	37.0	1,425	61.5	
One	29,672	24.4	460	21.3	
Two or more	52,510	38.7	333	17.2	
Smoking status					<0.001
Never smoker	67,551	56.0	1,915	87.2	
Past smoker	34,176	26.1	172	7.9	
Current smoker	22,617	17.7	126	4.8	
Alcohol use					<0.001
Never drinker	18,199	14.8	1,057	49.4	
Former drinker	19,942	14.4	112	5.5	
Current drinker	85,464	69.9	1030	44.1	
Region					<0.001
Northeast	22,515	19.0	490	24.4	
Midwest	33,088	27.5	385	16.8	
South	39,520	33.7	727	32.1	
West	29,494	19.8	616	26.7	
NHIS year					0.002
2012	20,767	17.0	404	13.8	
2013	20,119	16.5	394	14.4	
2014	22,360	16.6	389	16.2	
2015	20,359	16.5	394	17.3	
2016	22,727	16.7	341	20.4	
2017	18,285	16.7	296	18.0	

Based on 124,617 NHWs and 2,218 Asian Indians (age ≥18 years); cross-sectional data of NHIS participants (Asian Indians or NHWs), from multiple years (2012 through 2017), who participated in the sample adult core and did not have missing data on the PD scale. Numbers may not add up to the total in each group due to missing data for marital status, education, employment, poverty status, health insurance, physical activity, BMI, smoking status, and alcohol use.

BMI, body–mass index; K6, six-item Kessler; FPL, Federal Poverty Level; NHIS, National Health Interview Survey; NHWs, non-Hispanic Whites; PD, psychological distress.

Table 2 describes differences in demographics, lifestyle, socioeconomic status, behavioral characteristics, and health status by the prevalence of moderate-serious psychological distress.

**Table 2.** Description of Sample by the Prevalence of Moderate–Serious Psychological Distress in Adults (≥18 Years) Using the National Health Interview Survey, 2012–2017

	Moderate–serious PD		No moderate–serious PD		*p*
	*N* (26,094)	Wt. %	*N* (100,741)	Wt.%	
Sex					<0.001
Women	15,616	22.2	52,898	77.8	
Men	10,478	17.1	47,843	82.9	
Age in years					<0.001
18 to 39	8,722	21.8	29,726	78.2	
40 to 49	4,242	21.4	14,116	78.6	
50 to 64	7,705	20.2	27,061	79.8	
≥65	5,425	14.6	29,838	85.4	
Race/ethnicity					<0.001
NHWs	25,827	19.9	98,790	80.1	
Asian Indians	267	11.0	1,951	89.0	
Marital status					<0.001
Married	11,250	16.7	55,902	83.3	
Widow, separated, or divorced	8,689	25.7	26,049	74.3	
Never married	6,115	24.3	18,596	75.7	
Education					<0.001
Less than high school	3,331	29.8	7,574	70.2	
High school	7,134	22.2	24,188	77.8	
Some college	9,147	21.7	31,511	78.3	
College	6,404	13.8	37,238	86.2	
Poverty status					<0.001
<100% FPL	5,317	38.4	8,314	61.6	
100 to <200%	6,005	30.4	13,849	69.6	
200 to <400%	6,982	20.6	27,599	79.4	
≥400%	6,360	13.1	42,413	86.9	
Employment					<0.001
Employed	12,420	16.3	59,702	83.7	
Unemployed	13,668	24.8	40,994	75.2	
Health insurance					<0.001
Insured	22,689	18.7	92,898	81.3	
Uninsured	3,340	29.9	7,564	70.1	
Physical activity/exercise					<0.001
Daily exercise	1,584	17.4	7,147	82.6	
Weekly	7,491	15.7	38,261	84.3	
Monthly, yearly, or never	15,646	22.1	52,867	77.9	
Unable to exercise	1,185	43.2	1,682	56.8	
Race-adjusted BMI					<0.001
Underweight and normal	8,815	18.9	36,278	81.1	
Overweight	7,827	17.7	34,316	82.3	
Obese	8,705	23.1	27,247	76.9	
No. of chronic diseases					<0.001
No	6,686	14.9	37,158	85.1	
One	5,849	18.8	24,283	81.2	
Two or more	13,558	25.0	39,285	75.0	
Smoking status					<0.001
Never smoker	11,559	45.8	57,907	59.3	
Past smoker	6,847	24.8	27,501	26.0	
Current smoker	7,631	29.2	15,112	14.5	
Alcohol use					
Never drinker	3,572	14.2	15,684	15.8	<0.001
Former drinker	5,292	18.7	14,762	13.2	
Current drinker	17,030	66.4	69,464	70.2	
Region					<0.001
Northeast	4,583	17.8	18,422	82.2	
Midwest	6,738	20.4	26,735	79.6	
South	8,131	19.1	32,116	80.9	
West	6,642	21.6	23,468	78.4	
NHIS year					<0.001
2012	3,727	16.6	17,444	83.4	
2013	4,443	20.6	16,070	79.4	
2014	4,466	18.3	18,283	81.7	
2015	4,417	20.5	16,336	79.5	
2016	4,914	20.4	18,154	79.6	
2017	4,127	22.0	14,454	78.0	

Based on 124,617 NHWs and 2,218 Asian Indians (age ≥18 years); NHIS participants (Asian Indians or NHWs), from multiple years (2012 through 2017), who participated in the sample adult core and did not have missing data on the PD scale. Statistically significant differences in characteristics by Asian Indian and NHW status were tested with Rao–Scott chi-square tests. Numbers may not add up to the total in each group due to missing data for marital status, education, employment, poverty status, health insurance, physical activity, BMI, smoking status, and alcohol use.

FPL, Federal Poverty Level.

### Unadjusted and adjusted associations of Asian Indian ethnicity with PD

Based on the K6 scale, 19.7% of the sample reported moderate–serious PD, whereas 3.4% of the sample reported serious PD. The ORs and adjusted odds ratios (AORs) from multivariable logistic regression determining the association of race/ethnicity with moderate–serious PD are shown in Table 3. In the unadjusted model (model 1), Asian Indians were less likely to have moderate–serious PD compared with NHWs (OR=0.50; 95% CI: 0.42–0.58).

**Table 3.** Unadjusted and Adjusted Odds Ratios and 95% Confidence Intervals from Multivariable Logistic Regression Determining the Association of Race/Ethnicity with Moderate–Serious Psychological Distress in Adults (≥18 years) Using the National Health Interview Survey, 2012–2017

Model 1: unadjusted	Moderate–serious PD		
Racial/ethnic categories	UOR	95% CI	Sig
Asian Indians	0.50	0.42–0.58	^***^
NHWs (reference group)			

Model 2: controlling for sex and age	Moderate–serious PD		
Racial/ethnic categories	AOR	95% CI	Sig
Asian Indians	0.46	0.39–0.54	^***^
NHWs (reference group)			

Model 3: controlling for sex, age, and education			
Racial/ethnic categories	AOR	95% CI	Sig
Asian Indians	0.57	0.49–0.68	^***^
NHWs (reference group)			

Model 4: controlling for sex, age, education, marital status, socioeconomic status, health insurance, and physical activity			
Racial/ethnic categories	AOR	95% CI	Sig
Asian Indians	0.54	0.46–0.64	^***^
NHWs (reference group)			

Model 5: controlling for sex, age, education, marital status, socioeconomic status, health insurance, physical activity, race-adjusted BMI, number of chronic diseases, smoking, and alcohol use status			
Racial/ethnic categories	AOR	95% CI	Sig
Asian Indians	0.72	0.59–0.82	^***^
NHWs (reference group)			

Based on 124,617 NHWs and 2,218 Asian Indian adults (age ≥18 years); cross-sectional data of NHIS participants (Asian Indians or NHWs), from multiple years (2012 through 2017), who participated in the sample adult core and did not have missing data on the PD scale. Statistically significant differences in characteristics by Asian Indian and NHW status were tested with Rao–Scott chi-square tests.

^*^ 0.01 ≤ *p*<0.05; ^**^0.001 ≤ *p*<0.01; and ^***^p<0.001.

AOR, adjusted odds ratio; UOR, unadjusted odds ratio; CI, confidence interval.

After controlling for biological factors, the adjusted odds ratio of moderate–severe PD in Asian Indians was further reduced (AOR=0.46; 95% CI: 0.39–0.54). Education was highly protective against PD; when controlling for education in model 3, the difference in the likelihood of moderate–serious PD in Asian Indians reduces, but still remains significantly lower in Asian Indians compared with NHWs (AOR=0.57; 95% CI: 0.49–0.68).

In model 5, after controlling for all the known risk and protective factors and behavioral characteristics (smoking and alcohol use), moderate–serious PD among Asian Indians remained statistically significantly lower compared with NHWs (AOR=0.72; 95% CI: 0.61–0.85). In the fully adjusted model 6 (not shown in Table 3), Asian Indians were significantly less likely to have PD than NHWs (AOR=0.7; 95% CI: 0.59–0.82).

## Discussion

In this study, we examined the association of Asian Indian ethnicity with PD by comparing Asian Indians with NHWs. Our study shows that even after controlling for the relevant risk and protective factors related to PD, Asian Indians showed a lower prevalence of the disease than NHWs. In our study, the prevalence of moderate–serious PD was 19.7%, similar to the 18.2% combined moderate and serious PD reported separately by Mojtabai and Jorm using NHIS data from 2001 to 2012.^6^

We also found the prevalence of serious PD from 2012 to 2017 at 3.4%, which was similar to that reported by other national studies using NHIS data. For instance, Mojtabai and Jorm and Tomitaka et al. reported serious PD at 3.1% from 2001 to 2012,^6,7^ and CDC reported serious PD at 2.6% to 3.6% from 1997 to 2017.^68^

The multivariable logistic regression analysis showed that Asian Indians were less likely to report moderate–serious PD compared with NHWs. The prevalence of moderate–serious PD was 11% in Asian Indians compared with 19.9% in NHWs. As this is the first study to examine PD among Asian Indians in the United States, we do not have any published studies for comparison.

However, our findings of PD in Asian Indians are consistent with those of other Asian racial groups in the United States. For instance, Kim et al.,^21^ the CDC,^68^ and Bratter and Eschbach^24^ reported a lower PD score in Asian Americans than NHWs. The major difference between these studies and our study is that they either incorporated all Asian races/ethnicities in one group or did not include Asian Indians in their studies.

The lower prevalence in Asian Indians could be explained by high socioeconomic status, which acts as a protective factor against mental health problems. Consistent with published literature, Asian Indians had a favorable mental health profile.^69^ For instance, Asian Indians reported higher levels of protective factors such as education, income, employment, and marital support.

Asian Indians also showed an overall lower prevalence of chronic diseases than NHWs. However, Asian Indians reported high prevalence of a few chronic diseases such as diabetes (not reported separately in the article) and obesity (48% compared with 28% in NHWs). These findings probably explain the suppression effect in regression model 5.

We observed that even after controlling for established protective and risk factors, Asian Indians were less likely to have moderate–severe PD. We speculate that this can be explained by many factors that we did not control for in the study. For instance, Asian Indians have high expectations regarding education and success, collectivism, and a strong cultural continuity in their community.^70–73^ Asian Indians also preserve a strong ethnic identity and traditional family structure, pay more attention to parenting, and reinforce their high achievements on children.^70,71,74–76^

Moreover, Asian Indians have a dense social network and derive high social support from their family, relatives, and community.^77^ These strong cultural/ethnic identity and social support characteristics among the Asian Indian community can plausibly act as a buffer against PD. Immigration is another factor that could contribute to the lower distress in Asian Indians.

In our study, most Asian Indians (91.1% vs. 5.20%) were born outside the United States, which could indicate a healthy immigrant effect related to selective migration and a healthier state of new immigrants.^71,78,79^

We cannot rule out systematic underreporting of mental illness in Asian Americans. In general, Asian Americans are ashamed and embarrassed about mental illness and seeking mental health treatment.^80–82^ Cultural norms in Asian communities often lead to underreporting of health conditions in self-reported interviews.

In the Asian Indian community, mental health issues are often justified under the religious and spiritual framework. Mental hardship is considered God's will, a spiritual curse, or as repercussions of sins.^83–85^ Mental illness is viewed as a sign of weakness, and it is believed that the disclosure of mental illness will cause rejection from friends and community members and bring disgrace to the family.^83,84^ This results in Asian Indians not seeking professional mental health treatment and instead relying on religious/spiritual leaders and family members to discuss mental health issues.^83,84^

Individuals from Asian cultures also face other challenges such as language barriers and difficulties in navigating complex health care delivery systems for recent immigrants. Studies based on claims data have shown a higher rate of multimorbidity among Asian Americans than in NHWs compared with self-reports.^86,87^

Findings in our study should be interpreted in the context of their limitations. The study's major strengths are that we included nationally representative data for a period of 6 years and comprehensive lists of independent variables were used to test our study objective.

Limitations include cross-sectional data from NHIS; hence causal relationships could not be established. The data in NHIS are self-reported; thus, the findings are subject to recall bias and underreporting, as discussed. Finally, due to the low sample size of Asian Indians, it was not feasible to separately analyze moderate and serious PD.

## Conclusions

Our study concludes that Asian Indians are less likely to report PD compared with NHWs. The lower prevalence of distress is attributed to higher socioeconomic status and lower prevalence of chronic diseases. We recommend that mental health practitioners and future researchers should understand the distinctive characteristics and diversity of Asian Americans and other racial minority groups in the United States to better serve these populations.

## Abbreviations Used

AOR: adjusted odds ratio
BMI: body–mass index
CI: confidence interval
K6: six-item Kessler
NHIS: National Health Interview Survey
NHWs: non-Hispanic Whites
OR: odds ratio
PD: psychological distress
UOR: unadjusted odds ratio

## Appendix

**Appendix Table A1.** Description of Sample Characteristics of Non-Hispanic White and Asian Indian Adults (≥18 Years) Using the National Health Interview Survey, 2012–2017

Sample characteristics	N=126,835	Wt. % 100.0
Moderate–serious PD (K6≥5)		
Moderate–serious PD	26,094	19.7
No PD	100,741	80.3
Serious PD (K6≥13)		
Serious PD	4,584	3.40
No PD	122,251	96.6
Sex		
Women	68,514	51.4
Men	58,321	48.6
Race/ethnicity		
Non-Hispanic Whites	124,617	98.0
Asian Indians	2,218	2.0
Age in years		
18 to 39	38,448	34.0
40 to 49	18,358	16.1
50 to 64	34,766	27.6
≥65	35,263	22.2
Marital status^a^		
Married	67,152	63.8
Widow, separated, or divorced	34,738	17.6
Never married	24,711	18.5
Education^a^		
Less than high school	10,905	8.2
High school	31,322	24.6
Some college	40,658	31.6
College	43,642	35.3
Poverty status^a^		
<100% FPL	13,631	8.3
100 to <200%	19,854	13.6
200 to <400%	34,581	26.8
≥400%	48,773	43.1
Employment^a^		
Employed	72,122	60.1
Unemployed	54,662	39.8
Health insurance^a^		
Insured	115,587	91.2
Uninsured	10,904	8.5
Physical activity/exercise^a^		
Daily exercise	8,731	7.0
Weekly	45,752	37.9
Monthly, yearly, or never	68,513	52.5
Unable to exercise	2,867	1.8
Race-adjusted BMI^a^		
Underweight and normal	45,093	35.6
Overweight	42,143	33.2
Obese	35,952	28.4
No. of chronic diseases		
None	43,844	37.5
One	30,132	24.3
Two	52,843	38.1
Smoking status^a^		
Never smoker	69,466	56.6
Past smoker	34,348	25.8
Current smoker	22,743	17.4
Alcohol use^a^		
Never drinker	19,256	15.5
Former drinker	20,054	14.2
Current drinker	86,494	69.4
Region		
Northeast	23,005	19.1
Midwest	33,473	27.3
South	40,247	33.6
West	30,110	20.0
National Health Interview Survey year		
2012	21,171	16.9
2013	20,513	16.5
2014	22,749	16.6
2015	20,753	16.5
2016	23,068	16.8
2017	18,581	16.7

^a^ Represents the missing data; marital status, smoking, and employment status have less than 0.4% missing data. Physical activity and alcohol use have 0.8% missing data, race-adjusted BMI has 2.9% missing data, and poverty status has 7.9% missing data.

BMI, body–mass index; FPL, Federal Poverty Level; K6, six-item Kessler; PD, psychological distress.
